# Improved Interpretation of Pulmonary Artery Wedge Pressures through Left Atrial Volumetry—A Cardiac Magnetic Resonance Imaging Study

**DOI:** 10.3390/jcdd11060178

**Published:** 2024-06-11

**Authors:** Gülmisal Güder, Theresa Reiter, Maria Drayss, Wolfgang Bauer, Björn Lengenfelder, Peter Nordbeck, Georg Fette, Stefan Frantz, Caroline Morbach, Stefan Störk

**Affiliations:** 1 Division of Cardiology, Department of Internal Medicine I, University Hospital Würzburg, 97080 Würzburg, Germany; reitert@dhm.mhn.de (T.R.); drayss_m@ukw.de (M.D.); bauer_w@ukw.de (W.B.); lengenfeld_b@ukw.de (B.L.); nordbeck_p@ukw.de (P.N.); frantz_s@ukw.de (S.F.); morbach_c@ukw.de (C.M.); stoerk_s@ukw.de (S.S.); 2 Department of Clinical Research & Epidemiology, Comprehensive Heart Failure Center, University Hospital Würzburg, 97078 Würzburg, Germany; fette_g@ukw.de; 3 Department of Cardiac Rhythm Disorders, German Heart Center Munich, 80636 Munich, Germany; 4 Service Center Medical Informatics (SMI), University of Würzburg, 97080 Würzburg, Germany

**Keywords:** pulmonary capillary wedge pressure, left ventricular end-diastolic pressure

## Abstract

Background: The pulmonary artery wedge pressure (PAWP) is regarded as a reliable indicator of left ventricular end-diastolic pressure (LVEDP), but this association is weaker in patients with left-sided heart disease (LHD). We compared morphological differences in cardiac magnetic resonance imaging (CMR) in patients with heart failure (HF) and a reduced left ventricular ejection fraction (LVEF), with or without elevation of PAWP or LVEDP. Methods: We retrospectively identified 121 patients with LVEF < 50% who had undergone right heart catheterization (RHC) and CMR. LVEDP data were available for 75 patients. Results: The mean age of the study sample was 63 ± 14 years, the mean LVEF was 32 ± 10%, and 72% were men. About 53% of the patients had an elevated PAWP (>15 mmHg). In multivariable logistic regression analysis, NT-proBNP, left atrial ejection fraction (LAEF), and LV end-systolic volume index independently predicted an elevated PAWP. Of the 75 patients with available LVEDP data, 79% had an elevated LVEDP, and 70% had concomitant PAWP elevation. By contrast, all but one patient with elevated PAWP and half of the patients with normal PAWP had concomitant LVEDP elevation. The Bland–Altman plot revealed a systematic bias of +5.0 mmHg between LVEDP and PAWP. Notably, LAEF was the only CMR variable that differed significantly between patients with elevated LVEDP and a PAWP ≤ or >15 mmHg. Conclusions: In patients with LVEF < 50%, a normal PAWP did not reliably exclude LHD, and an elevated LVEDP was more frequent than an elevated PAWP. LAEF was the most relevant determinant of an increased PAWP, suggesting that a preserved LAEF in LHD may protect against backward failure into the lungs and the subsequent increase in pulmonary pressure.

## 1. Introduction

Increased pulmonary arterial wedge pressure (PAWP) assessed in right heart catheterization (RHC) is a typical feature of left heart disease and, in the absence of mitral valve disease, a reliable proxy for both increased left atrial and left ventricular filling pressures [1].

While 12 mmHg is the accepted upper limit of normal for the PAWP [2], previous guidelines and consensus statements arbitrarily chose the higher PAWP threshold of >15 mmHg to distinguish post-capillary from pre-capillary pulmonary hypertension in patients with elevated pulmonary artery pressure [1]. Further, a PAWP threshold of ≥15 mmHg or a left ventricular end-diastolic pressure (LVEDP) of ≥16 mmHg at rest suffices to invasively confirm the diagnosis of heart failure (HF) with preserved ejection fraction (HFpEF) [3,4].

The correlation between PAWP and LVEDP in left-sided heart disease was repeatedly described as poor, even when LVEDP and PAWP were measured simultaneously [5,6,7]. In patients with underlying cardiac disease, LVEDP levels are frequently higher than PAWP levels for various reasons. An important exception are conditions with a high V-wave, such as atrial fibrillation or mitral valve regurgitation, where this association is seemingly inverse [8,9]. 

While LVEDP solely reflects the performance of the left ventricle, PAWP represents the sum of the hemodynamic interplay between left ventricular, left atrial, and pulmonary venous (dys-)function. Thus, despite elevated LVEDP levels, PAWP might still be within normal ranges if, for instance, left atrial integrity is preserved [8].

The purpose of this study was to characterize the morphological differences in standard cardiac magnetic resonance imaging (CMR) of the left atrium (LA), the left ventricle (LV), and the atrioventricular coupling between patients with heart failure and CMR-confirmed reduction of the left ventricular ejection fraction (LVEF) below 50% with and without PAWP or LVEDP elevation. 

## 2. Materials and Methods

### 2.1. Study Design and Patient Selection 

This was a retrospective analysis based on medical information retrieved from the dedicated electronic data warehouse of the University Hospital of Würzburg [10]. The system facilitates a customizable, in-depth search and can track patient information over time. For the current analyses, we identified patients treated by the Department of Internal Medicine of the University Hospital of Würzburg who had undergone RHC and CMR [11]. Patient data from multiple sources collected by the data warehouse were utilized, including discharge letters, International Classification of Diseases codes, diagnostic reports, and procedure codes [12]. Due to this study’s retrospective design and the pseudonymized search modus, ethical approval was waived by the local Ethics Committee. The data steward in charge of the data transfer via the data warehouse approved the data extraction for this study. This study was conducted in accordance with the Declaration of Helsinki. 

We identified 293 consecutive patients reporting symptoms of heart failure, for whom data were available on RHC and CMR, between January 2016 and January 2022. One hundred forty-five patients had an LVEF < 50% in CMR. Of those, 24 patients had to be excluded from analysis because the time period between CMR and RHC was longer than 14 days (*n* = 12), information on PAWP or mean PAP was missing (*n* = 8), or shunting conditions were evident (*n* = 4). Thus, the current analysis refers to 121 patients. 

Transthoracic echocardiography was performed according to practice guidelines [13] as part of the clinical routine during the hospitalization or an outpatient visit. The median time difference between echocardiography and RHC was two days (quartiles 1 and 6 days). CMR was performed on a 1.5 T Achieva or a 3.0 T Achieva DS scanner (Philips Healthcare, Best, The Netherlands). The median time between CMR and RHC investigations was three days (quartiles 1 and 5 days). To determine the ventricular volumes, a short-axis CINE stack was used to cover the ventricles from the apex to the valvular plane [14]. During the end-systolic and end-diastolic phases, the endomyocardial border was traced manually, with the papillary muscle being considered a part of the intracavitary volume. Right ventricular and left ventricular stroke volumes (SVs) were calculated by computing the difference between the end-diastolic (EDV) and end-systolic (ESV) volumes of either ventricle. LVEF was calculated by dividing the SV by the EDV and multiplying it by 100. Maximal LA volumes (LAVs) were determined at the end-systole of the LV (LAV_ES_) and minimal LAV at the end-diastole of the LV (LAV_ED_) with the area–length method [15] using the following formula:

0.848 × LA-area [4-chamber view] × LA-area [2-chamber view)]/(length [2-chamber view] + length [4-chamber])/2).

**Figure 1:**
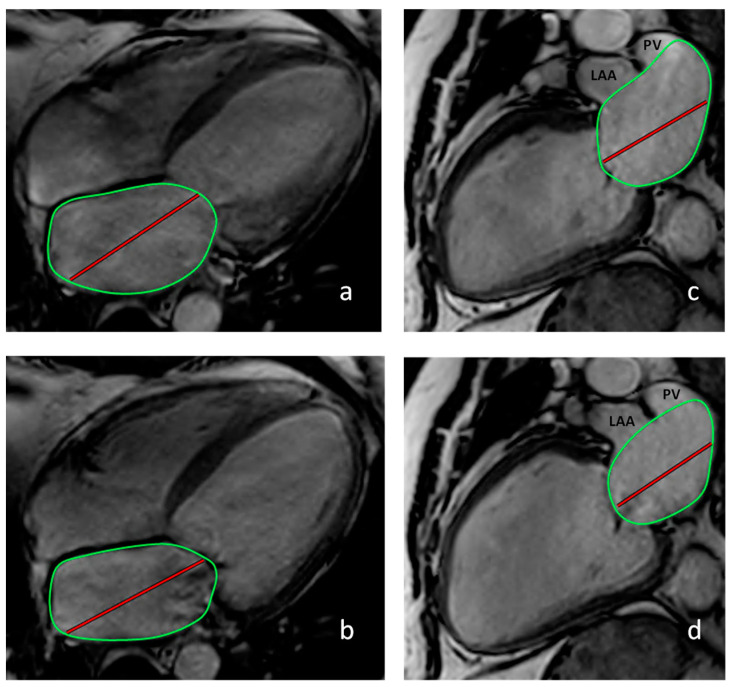
Calculation of the left atrial ejection fraction. Calculation of LA volume based on the 4-chamber (CH) view (**a**,**b**) and the 2-CH view (**c**,**d**). Abbreviations: LAA: left anterior appendage; PV: pulmonary vein. (**a**) End-systolic 4-CH view. (**b**) End-diastolic 4-CH view. (**c**) End-systolic 2-CH view (LAA and PV were excluded from the atrial area). (**d**) End-diastolic 2-CH view (LAA and PV were excluded from the atrial area; green circles correspond to the LA-area, red line to the LA-length).

The left atrial ejection fraction (LAEF) was calculated by subtracting maximal and minimal LAV divided by maximal LAV multiplied by 100 (100 × [(LAV_ES_ − LAV_ED_)/LAV_ES_]; Figure 1) [16]. As previously described, the left atrioventricular coupling index was calculated by dividing the LAV_ED_ by the LVEDV and expressed as a percentage [17]. RHC was performed according to standard recommendations [18], either alone or combined with coronary angiography using an Edwards Lifesciences Vigilance II™ monitor or the Schwarzer Cardiotek Evolution system. Cardiac output (CO) was measured using the thermodilution method [19]. In eight patients with missing CO values according to the thermodilution method, CO was estimated using the indirect Fick method, as suggested by Krakau [11]. 

The ABL80 FLEX CO-OX blood gas analyzer (Radiometer Medical ApS, Brønshøj, Denmark) was used to measure hemoglobin levels and oxygen saturation of mixed venous blood (PA-SO_2_). Arterial oxygen saturation (SaO_2_) was derived from finger pulse oximetry or measured invasively in patients with additional arterial catheterization. The formula of Dubois and Dubois was applied to calculate the body surface area (BSA) used for indexing volume measurement in CMR and cardiac output in RHC [20]. Data from RHC (hemodynamics and pressure tracings) were double-checked and entered manually by two cardiologists (GG and TR).

### 2.2. Definition of Heart Failure and Pulmonary Hypertension

All patients had signs or symptoms of heart failure. Heart failure (HF) was defined according to the HF guidelines of the European Society of Cardiology (ESC). When LVEF was reduced to ≤40%, HF with reduced ejection fraction (HFrEF) was diagnosed; when LVEF was <50% but >40% in CMR, HF with mildly reduced ejection fraction (HFmrEF) was diagnosed [4]. Patients with an LVEF ≥ 50% were excluded to provide morphological evidence of HF in all patients.

The 2022 ESC/ERS guidelines for pulmonary hypertension (PH) were used for defining pre-capillary PH (mean pulmonary artery pressure [PAP] > 20 mmHg plus mean PAWP ≤ 15 mmHg and pulmonary vascular resistance [PVR] > 2 Wood units) or post-capillary PH (mean PAP > 20 mmHg plus mean PAWP > 15 mmHg) [1,21]. Post-capillary PH was further divided into isolated post-capillary PH (if PVR was ≤2 WU) and combined post- and pre-capillary PH (if PVR was >2 WU) [1]. A PAWP or LVEDP level of >15 mmHg was defined as elevated.

### 2.3. Data Analysis

Data are reported as count (per cent), mean ± SD, or median (quartiles). Group comparisons were performed for nominal and ordinal parameters using Fisher’s exact test or chi-square test and for metric parameters using the Mann–Whitney U-test or Kruskal–Wallis test. The level of agreement between PAWP and LVEDP elevation was tested with the Cohen’s kappa statistic [22]. Univariable logistic regression was used to identify significant (*p* < 0.05) predictors of a PAWP > 15 mmHg. Multivariable logistic regression analysis was used to determine independent predictors of a PAWP > 15 mmHg. Variables with a high correlation (Pearson correlation coefficient > 0.8) were not included in the model. Statistical significance was assumed for all test procedures at a (two-sided) *p*-value of <0.05. All analyses were performed using IBM SPSS Statistics for Windows Version 29.

## 3. Results

Within the sample identified via the data warehouse search, 145 out of 266 (55%) patients with left-sided heart disease exhibited an LVEF < 50% in CMR. Because another 24 patients had incomplete information (see Section 2), the current analysis refers to 121 patients.

### 3.1. Baseline Characteristics

In the total sample (*n* = 121), the mean age was 63 ± 14 years, and 72% were men. The mean LVEF was 32 ± 10% in CMR; 89 patients (74%) had HFrEF, and 32 (26%) had HFmrEF. The predominant underlying cause of HF was dilated cardiomyopathy in the majority of patients (*n* = 58; 48%), followed by ischemic cardiomyopathy (*n* = 41; 34%) and valvular heart disease (*n* = 17; 14%), while the remaining patients (*n* = 4) suffered from amyloidosis, hypertrophic obstructive cardiomyopathy, restrictive cardiomyopathy, and an unknown cause. 

About half of the patients (*n* = 57; 47%) with a CMR-confirmed reduction of LVEF below 50% had a PAWP ≤ 15 mmHg. The New York Heart Association (NYHA) functional class was similar between patients with and without PAWP elevation, but patients with elevated PAWP had worse renal function (*p* = 0.042) and higher levels of N-terminal-prohormone of brain natriuretic peptide (NT-proBNP; *p* = 0.001; Table 1). 

The HF medication prescription was similar for patients with and without PAWP elevation. However, patients with elevated PAWP had a higher intake of loop diuretics (67 vs. 86%; *p* = 0.017; Table 1). In echocardiography, tricuspid annular plane systolic excursion (TAPSE; 18 vs. 15 mm; *p* = 0.002) was lower in patients with PAWP elevation. High-grade aortic stenosis but not mitral regurgitation was more common in patients with elevated PAWP (7 vs. 19%; *p* = 0.07; Table 1).

#### 3.1.1. Hemodynamic Differences in Patients with and without PAWP Elevation

Cardiac index tended to be lower (2.7 vs. 2.5 L/min/m^2^; *p* = 0.066), and LVEDP, mPAP, and mRAP were significantly higher in patients with elevated PAWP (Table 1, all *p* < 0.001). All but two patients with elevated PAWP (*n* = 62 of 64) had a diagnosis of post-capillary PH. The two patients with elevated PAWP who did not fulfill the criteria for post-capillary PH according to the 2022 guideline definition had a borderline elevation of the mPAP (both 20 mmHg). In 25 of 62 patients (40%), isolated post-capillary PH was diagnosed, and in 37 of 62 (60%) patients, combined post- and pre-capillary PH was diagnosed. In patients without PAWP elevation, pre-capillary PH was found in 12 patients (20%), with a median mPAP of 24 mmHg (quartiles: 22 and 27 mmHg). In 8 of those 12 patients, LVEDP levels were available; in 5 out of these 8 patients (63%), LVEDP levels were >15 mmHg. 

#### 3.1.2. Cardiac Magnetic Resonance Imaging

Patients with PAWP elevation had lower LAEF, LVEF, and RVEF in CMR (all *p* < 0.05). They also had larger cardiac cavities (both atria and ventricles, all *p* < 0.05). By contrast, the left atrioventricular coupling, LV, and RV stroke volume indices were not different (Table 1). 

### 3.2. Predictors of an Increased PAWP

Among the variables in Table 1 that showed a significant difference, correlates of an increased PAWP > 15 mmHg were sought using univariable logistic regression analysis (Table 2). Variables derived from RHC were not included due to their high interrelation with PAWP. Intake of loop diuretics, decreased TAPSE, worse renal function, increased NT-proBNP levels, worse LAEF, LVEF in CMR, and increased right and left heart sizes were associated with an elevated PAWP (Table 2). 

In multivariable logistic regression, NT-proBNP, LAEF, and LVESVi emerged as independent predictors using the backward selection approach. If the forward selection method was used, only NT-proBNP and LAEF remained significant. 

### 3.3. Correlation of PAWP with LVEDP

LVEDP was additionally available in 75 of 121 patients. Of those, 16 patients (21%) had LVEDP ≤ 15 mmHg, and 59 patients (79%) had an elevated LVEDP > 15 mmHg. Elevation of PAWP and LVEDP levels differed significantly (Table 3; *p* < 0.001): While in patients with an elevated PAWP > 15 mmHg, LVEDP was >15 mmHg in all but one patient (41/42; 98%), only 70% (41/59) of patients with elevated LVEDP had an increased PAWP > 15 mmHg. The single patient with a PAWP elevation without concordant LVEDP elevation had an LVEDP level of exactly 15 mmHg. 

**Figure 2:**
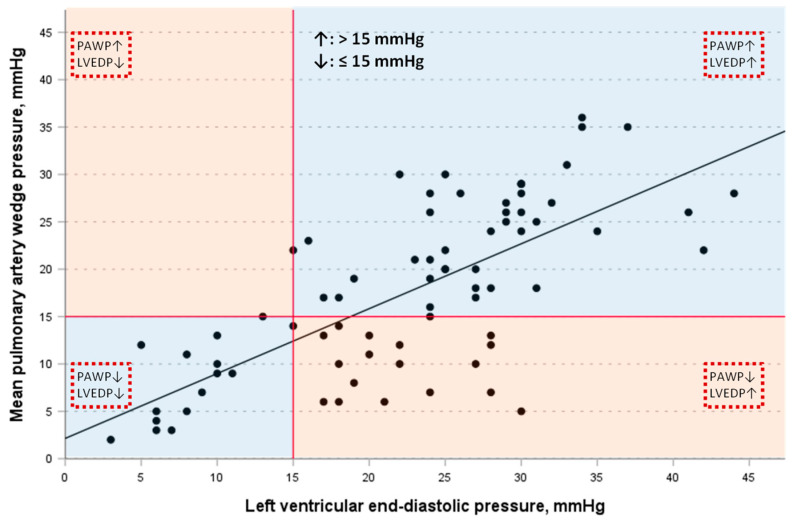
Scatter plot of left ventricular end-diastolic pressure (LVEDP) and mean pulmonary artery wedge pressure (PAWP) showing consistent (blue areas, both LVEDP and PAWP either non-elevated or elevated) and discrepant (rose areas, either LVEDP or PAWP elevated) associations, with the best-fit regression line for PAWP (PAWP = 2.14 + 0.68 × LVEDP).

Further, in patients with normal PAWP levels, LVEDP was elevated in more than half of the cases (18 out of 33 patients; 55%). PAWP correlated closely with LVEDP (Figure 2; Pearson correlation coefficient r = 0.71, 95% CI 0.58–0.81, *p* < 0.001, R^2^ = 0.504). The correlation was higher in patients with an LVEDP ≤ 15 mmHg (r = 0.80, 95% CI 0.51–0.93, *p* < 0.001) than in patients with an LVEDP > 15 mmHg (r = 0.56, 95% CI 0.36–0.71, *p* < 0.001). Cohen’s kappa statistic as a measure of agreement between elevated PAWP and elevated LVEDP > 15 mmHg was modest (0.46, 95% CI 0.27–0.64, *p* = 0.001). 

### 3.4. Linear Regression Models

Simple linear regression with PAWP being the dependent variable and LVEDP being the independent variable and vice versa was constructed (both associations, *p* < 0.001). 

The best-fit line of the regression equation for PAWP on LVEDP is shown in Figure 2 (PAWP = 2.14 + 0.68 × LVEDP). The regression equation for LVEDP on PAWP was LVEDP = 9.69 + 0.74 × PAWP.

**Figure 3:**
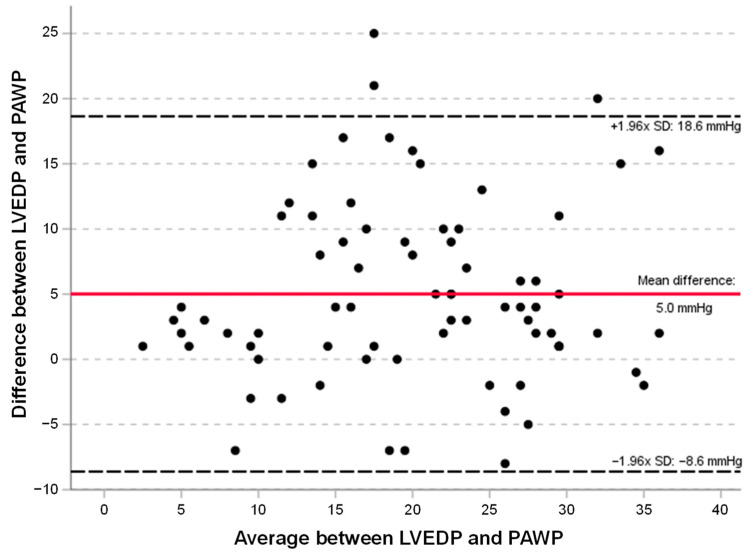
Bland–Altman plot comparing left ventricular end-diastolic pressure (LVEDP) with the mean pulmonary artery wedge pressure (mPAWP).

The Bland–Altman plot (Figure 3) revealed a systematic bias of 5.0 mmHg (with LVEDP on average higher than PAWP levels) and wide limits of agreement between mPAWP and LVEDP (−8.6; 18.6 mmHg). Of note, in patients with an LVEDP ≤ 15 mmHg, the mean difference between LVEDP and PAWP was −0.13 (SD ± 3.4) mmHg, and the median difference was 1 [quartiles −3; 2] mmHg vs. a mean difference of 6 (SD ± 7.0) mmHg and a median difference of 5 [quartiles 2; 11] mmHg in patients with an elevated LVEDP > 15 mmHg (*p* < 0.001). 

**Figure 4:**
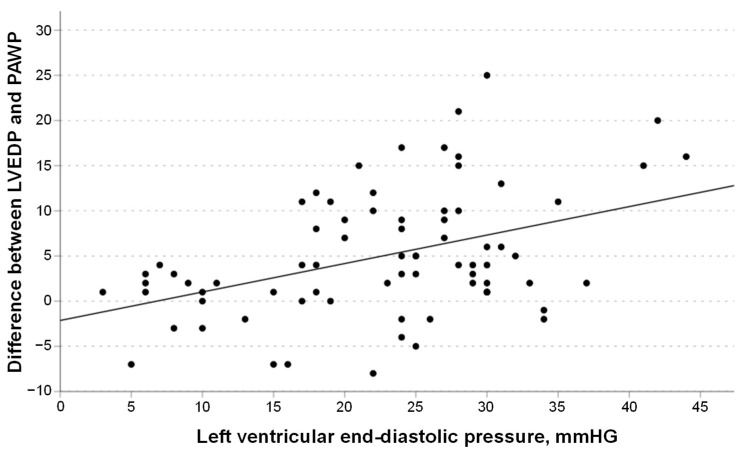
Correlation between the difference in left ventricular end-diastolic pressure (LVEDP) and mean pulmonary artery wedge pressure (mPAWP) and LVEDP (best fit line y = 2.14 + 0.32 × LVEDP).

The difference between LVEDP and PAWP correlated positively with increasing LVEDP levels (r = 0.42, 95% CI 0.21–0.59, *p* = 0.001, R^2^ = 0.177; Figure 4) and was >5 mmHg in 28 out of 75 patients (37%). All of these 28 patients had LVEDP levels > 15 mmHg.

The Bland–Altman plot of LVEDP and PAWP shows pairs of measurements from 75 patients. The ordinate refers to the difference between the LVEDP and PAWP. The abscissa refers to the mean between LVEDP and PAWP ([LVEDP + PAWP]/2). The red line indicates mean bias, and the dotted lines indicate the upper and lower borders of the 95% limits of agreement. 

### 3.5. Characteristics of Patients with Elevated LVEDP

Table 4 shows differences between patients with increased LVEDP with and without concurrent PAWP elevation. All variables in Table 1 were tested; only variables with significant differences and all CMR variables are shown. Patients with increased LVEDP and additional PAWP elevation had lower TAPSE levels (*p* = 0.015), lower CI, and higher PVR and right-sided pressure levels (mPAWP, mPAP, mRAP, LVEDP; all *p* < 0.05). Five patients with LVEDP elevation and normal PAWP fulfilled the criteria of pre-capillary PH. All but one patient with concomitant elevation of PAWP and LVEDP fulfilled the criteria of post-capillary PH (i.e., 40 out of 41; of those 27 patients (66%) had combined, and 13 patients (34%) had isolated post-capillary PH). The patient not fulfilling the criteria for post-capillary PH according to the 2022 guideline definition had a borderline increased mPAP of 20 mmHg.

In CMR, LV ejection fraction, LVEDD, and LV volumes were not different between patients with an elevated LVEDP and a PAWP ≤ or >15 mmHg (all *p* > 0.5; Table 4). However, indices of the LA were different, with worse LAEF and a trend towards larger end-diastolic volumes in patients with increased LVEDP and PAWP levels (Table 4). 

## 4. Discussion

In patients with left-sided heart disease and CMR-confirmed LVEF < 50%, only half of the cases had elevated mean PAWP, but about 80% had elevated LVEDP. More than half of patients with normal PAWP had elevated LVEDP levels, and all but one patient with an elevated PAWP > 15 mmHg had additional elevation of LVEDP. Independent predictors of PAWP elevation were NT-proBNP levels, LAEF, and LVESVi. In patients with elevated LVEDP, the only difference between patients with and without additional PAWP elevation in CMR was worse LAEF, suggesting that in left-sided heart disease, a preserved LAEF may protect against backward failure into the lungs and the subsequent increase in pulmonary pressure. 

Atrial enlargement and atrial fibrillation (a sequel of atrial enlargement) have been repeatedly described as determinants of PAWP elevation in left heart disease [7,9,23]. Garg et al. showed that PAWP levels could even be predicted non-invasively by a CMR-derived regression formula, including left atrial volume and left ventricular mass at rest [24,25] and after stress testing [26]. 

In our study, worse LAEF, increased NT-proBNP levels, and LV enlargement, but not atrial fibrillation or the recently proposed marker left atrioventricular coupling index (LACi), were independent predictors of PAWP elevation. NT-proBNP is highly correlated with LA and LV size and function [27,28]. An increase in LACi was an essential prognosticator of cardiovascular events, heart failure, and atrial fibrillation in the Multi-Ethnic Study of Atherosclerosis (MESA) [17,29,30] and in patients with acute myocardial infarction [31]. A clear explanation for the divergent relevance of LACi in our cohort has yet to be defined. However, compared to the mentioned study populations, the patients in our study had significantly worse LAEF and worse ventricular function. 

Under physiological circumstances, the pulmonary capillary bed, pulmonary veins, LA, and LV form a coherent unit in the end-diastole, with equal measurements of mPAWP, mean LA pressure, and LVEDP [1]. Consistently, in our study, the mean difference between LVEDP and PAWP in patients with a normal LVEDP (≤15 mmHg) was close to zero (mean −0.13 mmHg; SD ± 3.4). 

The mean PAWP can, therefore, be used to rule out left-sided heart disease in patients who do not have underlying cardiac disease accompanied by increased left-sided filling pressures but are, for instance, suspected of having pulmonary hypertension [1]. 

Notably, the approximations are less accurate under cardio-pathological conditions. In patients with left-sided heart disease, the agreements between PAWP and LA pressure and between PAWP and LVEDP [5] were repeatedly described as poor, with LVEDP exceeding PAWP levels by far [5,6,7,8]. This may lead to the misclassification of PH in patients with elevated mPAP into pre-capillary instead of post-capillary PH if LV filling pressures are not additionally assessed [8,32]. 

In our study, half of the patients with normal PAWP had elevated LVEDP levels, and the difference between LVEDP and PAWP was, on average, +5.0 (SD ± 7.0) mmHg. This order of magnitude compares well with other studies analyzing the difference between LVEDP and PAWP in patients with left-sided heart disease as high-grade aortic valve stenosis [7], but is higher than in patients with less severe heart disease or mixed populations with lung and/or heart diseases [6,23]. 

Technical issues may explain the discrepancies, such as non-simultaneous measurements of PAWP and LVEDP levels or incorrect placement of the Swan Ganz Catheter tip with the possibility of under- or over-wedging of the PAWP [18]. However, many studies suggest that the pathology of left-sided heart disease itself may cause disturbed associations [5,6]. 

The LV has different coping strategies to react to pathological conditions and maintain constant blood flow [33]. The increase in LVEDP is an expression of an abnormal ventricular pressure–volume relationship or worsening contractility found in patients with different forms of left-sided heart disease [33]. The pressure or volume load increase may not necessarily lead to backward failure, as the LA may respond to the augmented LV filling pressures with an increase in LA contractility [34]. Nevertheless, heart failure is a progressive disease, and atrial remodeling will likely develop over time. Such processes are typically accompanied by morphological and functional adaptations such as atrial dilatation, fibrosis, and electrical disturbances such as loss of sinus rhythm. Subsequently, atrial function worsens, and pulmonary pressure increases [34]. There is emerging evidence that restoration of sinus rhythm with catheter ablation, and thus amelioration of the atrial function, in patients with atrial fibrillation and (end-stage) heart failure confers prognostic benefit [35,36], contradicting the previous view that control of the ventricular response is sufficient to control heart failure in these circumstances [37]. 

Increased PAWP levels, whether invasively measured or non-invasively estimated, have repeatedly been linked to a worse prognosis in heart failure [38,39]. Studies have shown that an increase in PAWP is more closely associated with symptom burden and a worse prognosis than the elevation of LVEDP [40]. Since the elevation of PAWP in left-sided heart disease starts later or at a more advanced disease stage than the increase in LVEDP, these associations are not surprising and emphasize the importance of a preserved LAEF in patients with heart failure and a reduced ejection fraction. 

## 5. Limitations

This study has some limitations, as it is a retrospective single-center study with no standardized mode of data collection and a modest sample size. Further, including patients with CMR data likely selected healthier-than-average patients (e.g., not carrying CMR-incompatible cardiac devices, sufficient renal function, etc.). Only routine CMR data were used. Thus, atrial strain or atrial fibrosis were not assessed. Further, we focused on the left side of the heart. Therefore, information on the right atrium is limited. Additionally, PAWP and LVEDP levels were not measured simultaneously but sequentially, which is the standard in most catheter laboratories. However, this study’s central message, that the difference between LVEDP and PAWP is influenced by the left atrium, remains unaffected by all these shortcomings. 

## 6. Conclusions

In patients with left-sided heart disease and a reduced LVEF, the agreement between PAWP and LVEDP was high in patients with normal LVEDP but became worse with increasing LVEDP levels. PAWP elevation was less common than LVEDP elevation, and its occurrence depended on the size and EF of the LA. In patients undergoing RHC, a normal PAWP is, therefore, insufficient to reliably exclude left-sided heart disease.

## Figures and Tables

**Table 1:** Baseline characteristics.

	*n*	All	*n*	PAWP ≤ 15 mmHg	*n*	PAWP > 15 mmHg	*p*
Age, years	121	63 (55; 74)	57	62 (54; 74)	64	64 (56; 75)	0.58
Men, *n* (%)	121	87 (71.9%)	57	37 (64.9%)	64	50 (78.1%)	0.16
HFrEF, *n* (%)	121	89 (74%)	57	39 (68%)	64	50 (78%)	0.30
NYHA class ≥III, *n* (%)	121	62 (51.2%)	57	27 (47.4%)	64	35 (54.7%)	0.47
BMI, kg/m^2^	121	26.9 (23.7; 29.9)	57	25.8 (23.4; 29.6)	64	27.7 (24.0; 30.3)	0.13
DCM, %	121	58 (48%)	57	27 (47%)	64	31 (53%)	0.98
CAD, %	121	61 (50.4%)	57	27 (47.4%)	64	34 (53.1%)	0.59
Atrial fibrillation, *n* (%)	121	32 (26.4%)	57	15 (26.3%)	64	17 (26.6%)	1.00
Medication							
Betablocker, *n* (%)	121	112 (92.6%)	57	52 (91.2%)	64	60 (93.8%)	0.73
ACEi/ARB/ARNI, *n* (%)	121	113 (93.4%)	57	55 (96.5%)	64	58 (90.6%)	0.28
MRA, *n* (%)	121	78 (64.5%)	57	36 (63.2%)	64	42 (65.6%)	0.85
Loop diuretics, *n* (%)	121	93 (76.9%)	57	38 (66.7%)	64	55 (85.9%)	**0.017**
Laboratory							
eGFR, mL/min/1.73 m^2^	121	69 (55; 81)	57	72 (60; 86)	64	67 (49; 78)	**0.042**
Hemoglobin, g/dL	121	14.0 (12.6; 14.9)	57	14.1 (12.8; 15.6)	64	13.9 (12.5; 14.8)	0.16
NT-proBNP, pg/mL	98	3994 (1218; 8379)	46	1733 (910; 5088)	52	7104 (2276; 14,073)	**<0.001**
Echocardiography							
Aortic stenosis °III, *n* (%)	119	16 (13.3%)	55	4 (7.3%)	64	12 (18.8%)	0.07
Mitral regurgitation °III, *n* (%)	119	16 (13.4%)	55	6 (10.7%)	64	10 (15.6%)	0.59
TAPSE, mm	114	17 (14; 20)	51	18 (15; 21)	63	15 (12; 19)	**0.002**
Right heart catheterization							
Cardiac output, L/min	121	4.9 (4.1; 5.8)	57	4.9 (4.4; 5.8)	64	5.0 (3.7; 5.9)	0.43
Cardiac index, L/min/m^2^	121	2.6 (2.3; 3.0)	57	2.7 (2.4; 3.1)	64	2.5 (1.9; 2.9)	0.066
PVR, Wood units	121	2.0 (1.3; 2.9)	57	1.6 (1.3; 2.3)	61	2.3 (1.3; 3.9)	**0.027**
LVEDP, mmHG	75	24 (17; 29)	33	17 (9; 22)	42	28 (24; 31)	**<0.001**
mPAWP, mmHg	121	17 (9; 25)	57	9 (6; 12)	64	24 (20; 28)	**<0.001**
mPAP, mmHG	121	25 (18; 37)	57	17 (14; 22)	64	37 (30; 41)	**<0.001**
mRAP, mmHG	118	7 (4; 12)	56	4 (2; 7)	62	10 (8; 13)	**<0.001**
mPAP > 20 mmHg	121	76 (62.8%)	57	16 (28.1%)	64	60 (93.8%)	**<0.001**
Pre-capillary PH, *n* (%)	121	12 (9.9%)	57	12 (21.1%)	64	0 (0.0%)	**0.020**
Post-capillary PH, *n* (%)	121	62 (50.8%)	57	0 (0.0%)	64	62 (96.9%)	**<0.001**
Cardiac magnetic resonance imaging							
LAEF, %	105	23 (14; 34)	47	31 (22; 40)	58	17 (10; 27)	**<0.001**
LAV_ED_, mL	105	85 (61; 120)	47	65 (38; 101)	58	95 (72; 132)	**0.001**
LAVi_ED_, mL/m^2^	105	43 (31; 60)	47	34 (21; 56)	58	48 (38; 70)	**<0.001**
LAV_ES_, mL	105	112 (78; 153)	47	88 (66; 134)	58	114 (100; 161)	0.005
LAVi_ES_, mL/m^2^	105	57 (42; 74)	47	47 (36; 72)	58	60 (50; 79)	**0.006**
LACi_ED_, %	105	33 (24; 50)	47	31 (20; 46)	58	34 (26; 54)	**0.11**
LVEF, %	121	30 (24; 41)	57	34 (27; 44)	64	28 (22; 38)	**0.025**
LVEDD, mm	121	66 (60; 73)	57	64 (59; 71)	64	68 (60; 76)	**0.027**
LVEDV, mL	121	253 (192; 313)	57	218 (167; 277)	64	276 (208; 346)	**0.002**
LVEDVi, mL/m^2^	121	131 (100; 158)	57	117 (88; 148)	64	143 (108; 172)	**0.012**
LVESV, mL	121	167 (116; 231)	57	149 (106; 201)	64	194 (124; 256)	**0.002**
LVESVi, mL/m^2^	121	87 (62; 120)	57	78 (55; 107)	64	98 (68; 130)	**0.008**
LV stroke volume, mL	121	74 (62; 91)	57	73 (59; 88)	64	77 (64; 94)	0.28
LVSVi, mL/m^2^	121	39 (33; 46)	57	39 (32; 46)	63	39 (33; 48)	0.83
RVEF, %	120	46 (36; 56)	56	53 (41; 61)	64	42 (34; 52)	**0.002**
RA area, mm^2^	120	25 (19; 29)	56	22 (18; 27)	64	27 (20; 31)	**0.012**
RVEDD, mm	121	33 (29; 38)	57	32 (28; 35)	64	34 (29; 39)	0.087
RVEDV, mL	120	158 (122; 205)	56	142 (99; 184)	64	184 (141; 236)	**0.001**
RVEDVi, mL/m^2^	120	84 (63; 105)	56	73 (57; 94)	64	89 (72; 114)	**0.003**
RVESV, mL	120	89 (51; 125)	56	61 (42; 110)	64	98 (66; 152)	<0.001
RVESVi, mL/m^2^	120	44 (27; 65)	56	33 (21; 60)	64	49 (35; 72)	**0.001**
RV stroke volume, mL	120	70 (58; 86)	56	68 (53; 85)	64	72 (61; 87)	0.28
RVSVi, mL/m^2^	120	37 (30; 45)	56	36 (28; 43)	64	37 (31; 45)	0.54

Significant values are in bold. Values are total numbers (and percentages of *n*) or medians (25th–75th percentile). The *p* values refer to Fisher’s exact test, Chi-square-rest, or Mann–Whitney U-test, as appropriate. ACEi/ARB/ARNI, angiotensin-converting enzyme inhibitor, angiotensin receptor blocker, angiotensin receptor–neprilysin inhibitor; CAD, coronary artery disease; DCM, dilated cardiomyopathy; GFR, glomerular filtration rate; HFrEF, heart failure with reduced ejection fraction; LA, left atrium; LACi_ED_, left atrioventricular coupling index; LAV_ED_, left atrial volume end-diastolic; LAVi_ED_, LAV_ED_ index; LAV_ES_, left atrial volume end-systolic; LAVi_ES,_ LAV_ES_ index; LV, left ventricular; LVEDD, LV end-diastolic diameter; LVEDV, LV end-diastolic volume; LVEDVi, LVEDV index; LVEF, LV ejection fraction; LVESV, LV end-systolic volume; LVESVi, LVESV index; LVSVi, LV stroke volume index, mPAP, mean pulmonary artery pressure; mPAWP, mean pulmonary arterial wedge pressure; mRAP, mean right atrial pressure; MRA, mineralocorticoid receptor antagonist; NYHA, New York Heart Association; NT-proBNP, N-terminal-prohormone of brain natriuretic peptide; PH, pulmonary hypertension; PVR, pulmonary vascular resistance; RA, right atrium; RVEF, right ventricular ejection fraction; RVEDD, right ventricular end-diastolic diameter; RVEDV, RV end-diastolic volume; RVEDVi, RVEDV index; RVESV, RV end-systolic volume; RVESVi, RVESV indexed; RVSVi, RV stroke volume index; TAPSE, tricuspid annular plane systolic excursion.

**Table 2:** Determinants of an elevated pulmonary artery wedge pressure.

Predictors of PAWP > 15 mmHg	Univariable	Multivariable
Loop diuretics, yes vs. no	3.06 (1.25; 7.47); *p* = 0.014	-
TAPSE, per mm	0.86 (0.78; 0.95); *p* = 0.002	-
NTproBNP, per 1000 pg/mL	**1.23 (1.10; 1.39); *p* < 0.001**	**1.18 (1.03; 1.36); *p* = 0.018**
GFR, per 10 mL/min/1.73 m^2^	0.79 (0.65; 0.95); *p* = 0.012	-
LAEF, per %	**0.93 (0.89; 0.96); p < 0.001**	**0.93 (0.88; 0.98); *p* = 0.004**
* LAV_ED_, per mL	1.02 (1.01; 1.03); *p* = 0.002	
* LAVi_ED_, per mL/m^2^	1.03 (1.01; 1.06); *p* = 0.001	
* LAV_ES_, per mL	1.01 (1.00; 1.02); *p* = 0.012	
LAVi_ES_, per mL/m^2^	1.02 (1.00; 1.04); *p* = 0.013	*-*
LVEF, per %	0.96 (0.93; 1.00); *p* = 0.038	-
** LVEDD, per mm	1.05 (1.00; 1.09); *p* = 0.029	-
** LVEDV, per mL	1.01 (1.00; 1.01); *p* = 0.002	
** LVEDVi, per mL/m^2^	1.01 (1.00; 1.02); *p* = 0.009	
** LVESV, per mL	1.01 (1.00; 1.01); *p* = 0.002	
LVESVi, per mL/m^2^	**1.01 (1.00; 1.02); *p* = 0.006**	**1.03 (1.00; 1.05); *p* = 0.036**
RA area, per mm^2^	1.06 (1.01; 1.12); *p* = 0.028	
RVEF, per %	0.96 (0.93; 0.99); *p* = 0.003	
*** RVEDV, per mL	1.01 (1.00; 1.02); *p* = 0.001	
*** RVEDVi, per mL/m^2^	1.02 (1.01; 1.03); *p* = 0.005	
*** RVESV, per mL	1.01 (1.01; 1.02); *p* < 0.001	
RVESVi, per mL/m^2^	1.03 (1.01; 1.04); *p* = 0.002	

Abbreviation as in Table 1. Univariable and multivariable logistic regression with PAWP > 15 mmHg as the dependent variable. Independent predictors are highlighted in bold. The multivariable analysis did not include variables marked with asterisks due to their high interrelation with * LAVi_ES_, ** LVESVi, or *** RVESVi (Pearson correlation coefficient > 0.8).

**Table 3:** Contingency table for PAWP and LVEDP elevation.

		LVEDP (mmHg)		
		≤15	>15	Total
PAWP (mmHg)	≤15	15	18	33 (44%)
	>15	1	41	42 (56%)
	Total	16 (21%)	59 (79%)	75 (100%)

**Table 4:** Characteristics of patients with available LVEDP and PAWP pairs.

	*n* = 18	LVEDP > 15 mmHg and PAWP ≤ 15 mmHg	*n* = 41	LVEDP > 15 mmHg and PAWP > 15 mmHg	*p*
Echocardiography					
TAPSE, mm	17	20 (15; 24)	41	16 (14; 19)	**0.015**
Right heart catheterization					
CI, L/min/m^2^	18	2.9 (2.6; 3.2)	41	2.5 (2.0; 2.8)	**0.015**
PVR, Wood units	18	1.7 (1.3; 2.4)	41	2.7 (1.6; 4.6)	**0.030**
LVEDP, mmHG	18	22 (18; 27)	41	28 (24; 31)	**<0.001**
mPAWP, mmHg	18	10 (7; 13)	41	25 (20; 28)	**<0.001**
mPAP, mmHG	18	19 (16; 22)	41	39 (31; 42)	**<0.001**
mPAP > 20 mmHg, *n* (%)	18	7 (38.9%)	41	39 (95.1%)	**<0.001**
Pre-capillary PH, *n* (%)	18	5 (27.8%)	41	0 (0.0%)	**<0.001**
Post-capillary PH, *n* (%)	18	0 (0.0%)	41	40 (97.6%)	**<0.001**
mRAP, mmHG	18	5 (3; 7)	41	10 (8; 13)	**<0.001**
Cardiac magnetic resonance imaging					
LAEF,%	15	35 (25; 43)	38	16 (10; 25)	**<0.001**
LAV_ED_, mL	15	76 (38; 101)	38	95 (73; 143)	0.063
LAVi_ED_, mL/m^2^	15	38 (20; 58)	38	51 (39; 73)	0.055
LAV_ES_, mL	15	112 (70; 134)	38	116 (100; 159)	0.20
LAVi_ES_, mL/m^2^	15	56 (41; 70)	38	62 (51; 85)	0.24
LACi_ED_, %	15	33 (14; 44)	38	35 (25; 59)	0.13
LVEF, %	18	29 (26; 36)	41	28 (24; 41)	0.88
LVEDD, mm	18	68 (64; 72)	41	69 (58; 78)	0.58
LVEDV, mL	18	258 (211; 294)	41	276 (195; 353)	0.54
LVEDVi, mL/mm^2^	18	135 (111; 155)	41	140 (100; 174)	0.73
LVESV, mL	18	179 (140; 206)	41	186 (116; 265)	0.66
LVESVi mL/mm^2^	18	94 (77; 113)	41	99 (66; 131)	0.77
LV stroke volume	18	74 (60; 87)	41	79 (65; 94)	0.32
LVSVi, mL/m^2^	18	39 (31; 49)	41	40 (35; 47)	0.68
RVEF, %	18	45 (35; 63)	41	44 (34; 53)	0.34
RA area, mm^2^	18	21 (19; 26)	41	27 (20; 31)	0.06
RVEDD, mm	18	34 (28; 40)	41	32 (28; 40)	0.77
RVEDV mL/m^2^	18	145 (121; 196)	41	181 (137; 237)	0.12
RVEDVi, mL/m^2^	18	76 (63; 96)	41	90 (75; 116)	0.11
RVESV, mL	18	74 (49; 130)	41	98 (64; 149)	0.13
RVESVi, mL	18	40 (24; 64)	41	49 (36; 72)	0.13
RV stroke volume, mL	18	69 (58; 93)	41	72 (62; 85)	0.84
RVSVi, mL/m^2^	18	36 (29; 45)	41	37 (31; 45)	0.81

Abbreviations as in Table 1. Unless indicated otherwise, values are *n* (%) or median (25th, 75th percentile). Patients with an LVEDP > 15 mmHg were selected and grouped into groups without and with concomitant elevation (PAWP ≤ or >15 mmHg). All *p* values refer to Fisher’s exact test or the Mann–Whitney U test as appropriate; *p* ≤ 0.05 values are marked in bold.

## Data Availability

The approval by the institution’s data protection officer does not allow the data to be made publicly available. In case of any inquiries regarding further data analyses, please contact the corresponding author of this study.
